# A robust two-way semi-linear model for normalization of cDNA microarray data

**DOI:** 10.1186/1471-2105-6-14

**Published:** 2005-01-21

**Authors:** Deli Wang, Jian Huang, Hehuang Xie, Liliana Manzella, Marcelo Bento Soares

**Affiliations:** 1 Biostatistics and Bioinformatics Unit, Comprehensive Cancer Center, the University of Alabama at Birmingham, Birmingham, AL 35294, USA; 2 Department of Statistics and Actuarial Science, and Program in Public Health Genetics, the University of Iowa, Iowa City, IA 52242, USA; 3 Department of Pediatrics, the University of Iowa, Iowa City, IA 52242, USA; 4 Departments of Biochemistry, Orthopaedics, Physiology and Biophysics, the University of Iowa, Iowa City, IA 52242, USA

## Abstract

**Background:**

Normalization is a basic step in microarray data analysis. A proper normalization procedure ensures that the intensity ratios provide meaningful measures of relative expression values.

**Methods:**

We propose a robust semiparametric method in a two-way semi-linear model (TW-SLM) for normalization of cDNA microarray data. This method does not make the usual assumptions underlying some of the existing methods. For example, it does not assume that: (i) the percentage of differentially expressed genes is small; or (ii) the numbers of up- and down-regulated genes are about the same, as required in the LOWESS normalization method. We conduct simulation studies to evaluate the proposed method and use a real data set from a specially designed microarray experiment to compare the performance of the proposed method with that of the LOWESS normalization approach.

**Results:**

The simulation results show that the proposed method performs better than the LOWESS normalization method in terms of mean square errors for estimated gene effects. The results of analysis of the real data set also show that the proposed method yields more consistent results between the direct and the indirect comparisons and also can detect more differentially expressed genes than the LOWESS method.

**Conclusions:**

Our simulation studies and the real data example indicate that the proposed robust TW-SLM method works at least as well as the LOWESS method and works better when the underlying assumptions for the LOWESS method are not satisfied. Therefore, it is a powerful alternative to the existing normalization methods.

## Background

Microarray technology has become a useful tool for quantitatively monitoring gene expression patterns and has been widely used in functional genomics [1,2]. In a cDNA microarray experiment, cDNA segments representing a collection of transcripts and Expressed Sequence Tags (ESTs) are amplified by PCR and spotted in high density on glass microscope slides using a robotic system to produce cDNA microarrays. Each microarray contains thousands of such PCR products, named cDNA probes, which serve as reporters for the expression of the respective transcripts that represent the collection of genes or ESTs. The cDNA microarrays are queried in a co-hybridization assay using two fluorescently labeled biosamples derived from RNA obtained from the cell populations of interest. One sample is labeled with fluorescent dye Cy5 (red), and another with fluorescent dye Cy3 (green). Hybridization is assayed using a confocal laser scanner to measure fluorescence intensities, allowing simultaneous determination of the relative expression levels of all the genes represented on the slide [3].

A basic question in analyzing cDNA microarray data is normalization, the purpose of which is to remove systematic bias in the observed expression values by establishing a normalization curve across the whole dynamic range. A proper normalization method ensures that the normalized intensity ratios provide meaningful measures of relative expression levels. Normalization is needed because many factors, including different efficiency of dye incorporation, difference in the amount of RNA labeled between the two channels, uneven hybridizations, difference in the printing pin heads, among others, may cause bias in the observed expression values. Therefore, proper normalization is a critical component in the analysis of microarray data and can have important impact on higher level analysis such as detection of differentially expression genes, classification, and cluster analysis.

Many normalization methods have been proposed in the literature. The earliest normalization method for cDNA microarray data goes back to Chen et al. [4] who proposed a ratio-based method. Yang et al. [5] summarized several normalization methods for cDNA microarray data such as global normalization, dye-swap normalization, block-wise normalization, and scale normalization. They also proposed a locally weighted scatter plot smoothing (LOWESS [6]) method for intensity dependent normalization. Quackenbush [7] and Bilban et al. [8] provided good reviews on normalization methods for cDNA microarray data. Tseng et al. [9] proposed using a rank based procedure to first select a set of *invariant genes *that are likely to be constantly expressed and then carrying out LOWESS normalization using this set of genes. But as pointed out by Tseng et al., selected invariant genes may not cover the whole dynamic range of the expression values, and extrapolation is needed to fill in the gaps that are not covered by the invariant genes. Kepler et al. [10] also first estimated a set of "constantly expressed genes" and then used the LOWESS method. Wang et al. [11] proposed an iterative normalization method for cDNA microarray data by estimating a normalization coefficient and identifying control genes. Workman et al. [12] used array signal distribution analysis for a robust non-linear method of normalization. Park et al. [13] compared a number of normalization methods, including global, linear and LOWESS normalization methods. Wolfinger et al. [14] used a mixed model for normalization. They proposed a normalization model for normalization and a gene model for inference and these two models are related by the residual terms in the normalization model. A constant normalization factor assumption is needed in this method. Fan et al. [15] considered a Semi-linear-In-slide Model (SLIM) method using within-array replications. The SLIM method requires replication of a subset of the genes in an array. If the number of replicated genes is small, the expression values of the replicated genes may not cover the entire dynamic range or reflect spatial variation in an array. Fan et al. [16] generalized the SLIM method to account for across-array information, resulting in an aggregated SLIM, so that replication within an array is no longer required. Huang et al. [17] proposed a two-way semi-linear model (TW-SLM) for normalization of cDNA microarray data. They used the least squares method for estimating the normalization curves based on B-splines. This method does not require the assumptions required by the LOWESS normalization method, i.e. (i) a small fraction of genes are differentially expressed or (ii) there is symmetry in the expression levels of up- and down-regulated genes.

It is well known that the least squares method is not resistant to outliers which arise often in cDNA microarray experiments because of many sources of variations. In this paper, we propose a robust method for normalization in the framework of the TW-SLM. We conduct simulation studies and use a real cDNA microarray data set to compare the proposed method with the LOWESS normalization method.

## Results

### Simulation study

Simulation was conducted to compare the mean square errors (MSE) and biases of estimated gene expression levels between the proposed robust TW-SLM and LOWESS normalization methods, between the proposed method and the TW-SLM using OLS. The MSE for the *j*th gene is calculated as the following:


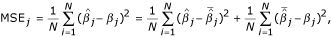


that is, 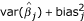, where *N *is the total number of replicates for each simulation, *J *is the number of unique genes, *β*_*j *_is the true gene expression level (base two log scale) for gene *j*, 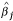 is the estimated value for *β*_*j*_, 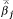 is the mean of 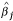 for *N *replicates, *j *= 1, 2,..., *J*, where *J *is the total number of genes. The data simulation procedure is based on the method proposed by Balagurunathan et al. [18]. In each simulation, we generated 10 slides with twelve blocks in each, and 500 genes in each block. We repeated 100 times for each simulation. The simulation procedure can be summarized in the following steps:

1. Simulate true signal intensity for each gene *j *using the exponential distribution with the mean of 3,000, i.e. *I*_*j *_~ exp(*λ *= 1/3000), for *j *= 1,..., *J*;

2. Simulate fluorescent intensity for the Cy5 channel and the Cy3 channel with the normal distribution, respectively. Suppose the coefficients of variation for intensity in the Cy5 channel and the Cy3 channel are *α*_*rj *_and *α*_*gj*_, respectively, then the fluorescent intensity on the two channels can be generated by the normal distribution with mean *I*_*j *_and standard deviations *α*_*rj*_*I*_*j *_and *α*_*gj*_*I*_*j *_for the red channel and the green channel, respectively. Let *R*_*j *_and *G*_*j *_represent simulated fluorescent intensity for the Cy5 channel and the Cy3 channel for gene *j*, respectively;

3. Simulate differentially expressed genes. Suppose *γ *× 100% genes are differentially expressed in the whole simulated gene set, then the ratio of the expression level for gene *j *can be generated by *t*_*j *_= 10^±*b *^with *b *~ *Beta*(1.7,4.8). The sign ± will determine if the gene is up- or down-regulated. The probability of the up-regulated genes within those *γ *× 100% differentially expressed genes is given as an input parameter. For the genes that are not differentially expressed, the *b *takes value zero;

4. Incorporate the *t*_*j *_into signal intensity of gene *j*. The *R*_*j *_and *G*_*j *_will be adjusted by adding the simulated expression ratio *t*_*j *_through the following formulae: 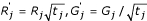 for *j *= 1,..., *J*;

5. Simulate a fluorescent system with the imperfect response characteristics. Due to various reasons, such as the unequal amount of mRNA for the two channels, different labeling efficiencies, or uneven laser powers at the scanning stage [18], actual intensity in the two channels are not exactly the same. More over, fluorescent intensity is not necessarily linearly related to the expression levels. Balagurunathan et al. proposed the following functional family,


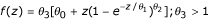


to distort the response characteristic functions of observed fluorescent intensity for the two channels, which are expressed as 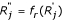 and 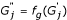, respectively. So four parameter values need to be determined for each channel before simulation. Different parameter values in the two channels will control the shape of the ratio vs. signal intensity plots (R-I plots);

6. Simulate background noise for each channel. The mean of background noise is determined by one input parameter: the signal to noise ratio (SNR) and the true mean of signal. The SNR is the ratio between the true mean of the signal and the true mean of background noise. The SNR controls variability of background noise. The normal distribution with a given mean value is used in simulating background noise. Variance of background noise will be controlled by the input parameters *α*_*br *_and *α*_*bg *_for the Cy5 channel and the Cy3 channel, respectively. These two parameters are the ratios between the mean and the standard deviations of background noise for the two channels, respectively. Simulated signal intensity for the two channels, 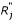 and 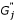, are adjusted by subtracting background noise in each channel. Let 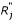 and 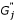 still denote background adjusted signal intensity for the two channels;

7. Add noise to the signal intensity for each channel. Finally, the signal intensity of each channel is generated by


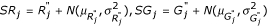


with 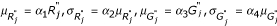, where *α*_1 _~ *U *(*a*_1_, *b*_1_), *α*_2 _~ *U *(*c*_1_, *d*_1_), *α*_3 _~ *U *(*a*_2_, *b*_2_), *α*_4 _~ *U *(*c*_2_, *d*_2_). The *a*_1_, *b*_1_, *c*_1_, *d*_1_, *a*_2_, *b*_2_, *c*_2_, *d*_2 _are given as input parameters to control variability of fluorescent signal intensity.

We simulated two situations, one is the no-dye bias case and another one is the shape case (dye bias exists). R-I plots of twelve blocks on one slide for two simulated cases are shown in Figures 2 and 3, respectively. We considered five different percentage levels of differentially expressed genes: 1%, 5%, 10%, 20%, and 40%. The ratio of the up-regulated genes to the down-regulated genes takes three values, i.e., 1:1, 3:1, and 9:1 at each percentage level of differentially expressed genes. In addition, based on the suggestion of a reviewer, we simulated an extreme case for the scenario in Figure 3, in which 70% genes are all up-regulated and the remaining ones are not differentially expressed.

The trend of MSEs and biases of estimated gene expression levels are similar between the robust TW-SLM and the LOWESS normalization methods across different levels of the ratios between the up-regulated genes and the down-regulated genes. This trend also exists in the extreme case. We present the results of the following two scenarios: (a) a 9:1 ratio between the number of the up-regulated genes and that of the down-regulated genes and, (b) the extreme case. Tables 1 and 3 present MSEs, Tables 2 and 4 show biases of estimated gene expression levels. MSEs and biases for the extreme case (70% of the genes are up-regulated) are presented in the bottom of Tables 3 and 4, which are displayed in Figures 6 and 7, respectively. The robust TW-SLM method has smaller means of MSEs than the LOWESS normalization method and the TW-SLM using OLS, respectively. Also the ranges of MSEs for the proposed method are also smaller than those using the LOWESS method and the TW-SLM with OLS, respectively.

Comparing the different robust weight functions, means of MSEs are slightly smaller using Tukey's weight function than that using Huber's weight function. These results are observed across different percentage levels of differentially expressed genes. Biases for estimated gene expression levels distributed similarly between the proposed method and the LOWESS normalization method. But the ranges of the biases for the proposed method are smaller than those of the LOWESS normalization method and the TW-SLM using OLS, respectively. These observations are true in both simulated situations.

The extreme case is an example where the proposed method does better than the LOWESS method (Tables 3 and 4, Figures 6 and 7). Estimates using the LOWESS method are downward biased in this case. This is what we would expect because the LOWESS method fits normalization curves through the majority of genes, which are mostly up-regulated here. In contrast, the TW-SLM method does not need the either of the two assumptions needed by the LOWESS method, neither of which is satisfied here.

The distributions of MSEs and biases between the TW-SLM using OLS and the LOWESS method are similar for cases where there is a relatively small percentage of differentially expressed genes. However, the TW-SLM with OLS performs better than the LOWESS when a larger proportion of genes are differentially expressed. It appears that the more deviation from the two assumptions required by the LOWESS, the better the TW-SLM performs. This trend is consistent with findings in our previous work [17].

### An example

In this section, a real data set was analyzed to compare consistency of the LOWESS normalization method and the proposed robust TW-SLM method. A collection of human placenta cDNAs comprising 7,042 clones was identified and used as the probe set for cDNA microarray fabrication in this study [19].

Three kinds of RNA samples were used which include: (i) a common reference RNA obtained by *in vitro *transcription from a pool of cDNAs in equal amount comprising the entire probe set (PS); (ii) the "Universal Human Reference RNA" from Stratagene, a pool of RNAs derived from 10 different cell lines; and (iii) human full-term placenta RNA. The original goal of the study was to evaluate the performance of the PS RNA as a reference RNA in comparison with that of Stratagene's universal reference RNA.

**Figure 1:**
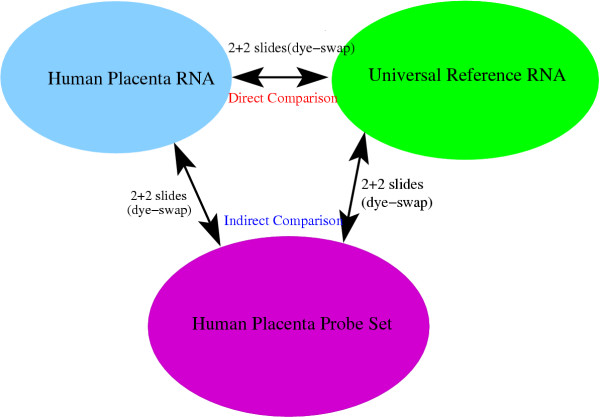
Study designs for cDNA microarray experiments among the human placenta, the universal reference, and the Probe Set.

**Figure 2:**
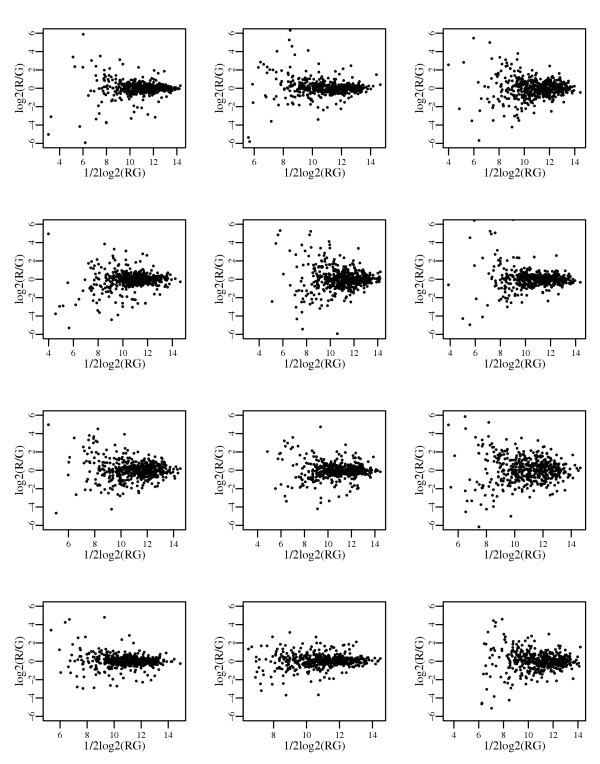
An example of R-l plots for twelve blocks of slide one with no-dye bias for two channels, 10% genes are differentially expressed.

**Figure 3:**
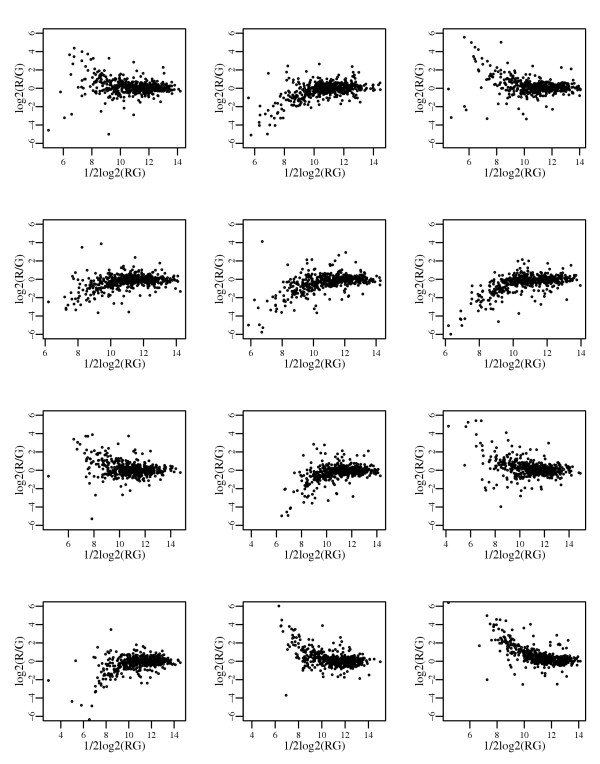
An example of shaped R-l plots for twelve blocks of slide one with the dye bias for two channels, 10% genes are differentially expressed.

In this study, the Universal Human Reference RNA and the human placenta RNA were treated as two experimental samples. The PS RNA was used as the reference against which the two other bio-samples were compared. In the simple direct comparison, gene expression values were obtained through direct hybridizations between the human placenta RNA and the Universal Human Reference RNA. In the indirect comparison using the PS set as the common reference, hybridizations were performed between the human placenta RNA and the PS reference RNA, and between the Universal Human Reference RNA and the PS reference RNA. The design of this experiment is depicted in Figure 1.

After hybridization, slides were scanned with the Axon instruments 4000B scanner. The 633 and 532 lasers are used for excitation of the Cy5 and Cy3 fluorophores, respectively. For each of the three types of hybridizations (i.e., the human placenta vs. the universal reference, the human placenta vs. the PS reference, and the universal reference vs. the PS reference), there are four slides, including two dye-swapped slides. Each clone was printed three times on different blocks on each slide. Background adjusted medians for the Cy5 and Cy3 channels were used as expression levels. We removed negative controls including "Human Cot1", "PolyA" and "Empty" in the analysis.

To evaluate the proposed method, we compare it with the LOWESS method by examining which method produces more consistent results between the direct comparison and the indirect comparison of human placenta and universal human reference RNA samples as described above (see also Figure 1). The rationale is that the results from the direct comparison design and the indirect comparison design should be similar, because the same RNA samples are compared in both designs, albeit the indirect comparison is through a third common reference. Therefore, a better normalization method is the one that yields more consistent results between the direct and indirect comparison experiments.

**Figure 4:**
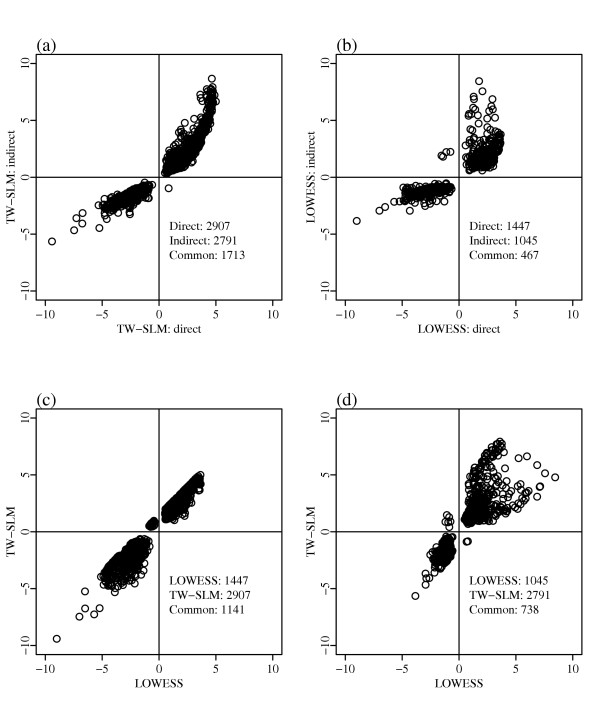
Consistency analysis based on cutoff p-value 10^-5^. Both *x *and *y *axes are estimated log intensity ratios. (a)-(b) between the direct design and the indirect design for the robust TW-SLM and the LOWESS normalization method, respectively; (c)-(d) between the LOWESS method and the robust TW-SLM for the direct design and the indirect design, respectively.

**Figure 5:**
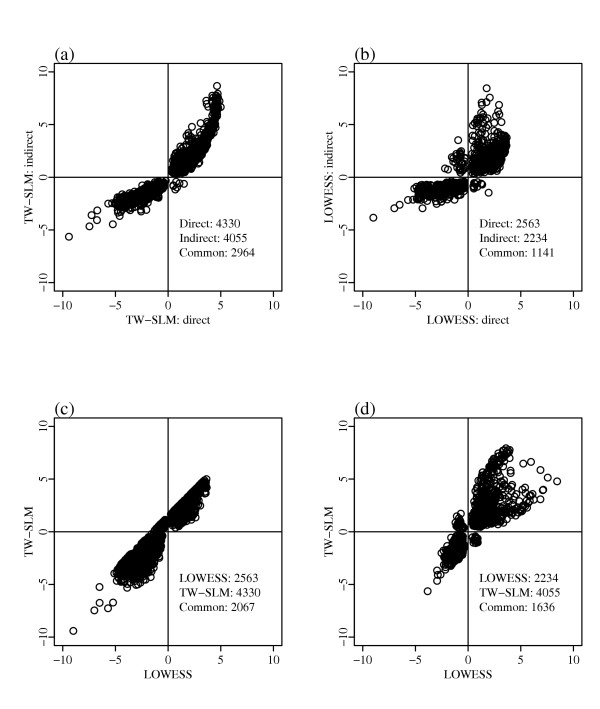
Consistency analysis based on cutoff p-value 10^-3^. Both *x *and *y *axes are estimated log intensity ratios. (a)-(b) between the direct design and the indirect design for the robust TW-SLM and the LOWESS normalization method, respectively; (c)-(d) between the LOWESS method and the robust TW-SLM for the direct design and the indirect design, respectively.

**Figure 6:**
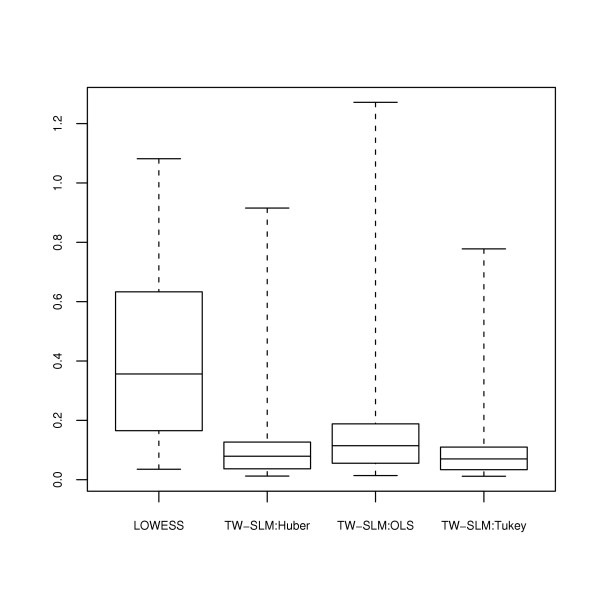
A boxplot for comparing mean square errors among normalization methods for the case that 70% genes are all up-regulated (Table 3).

**Figure 7:**
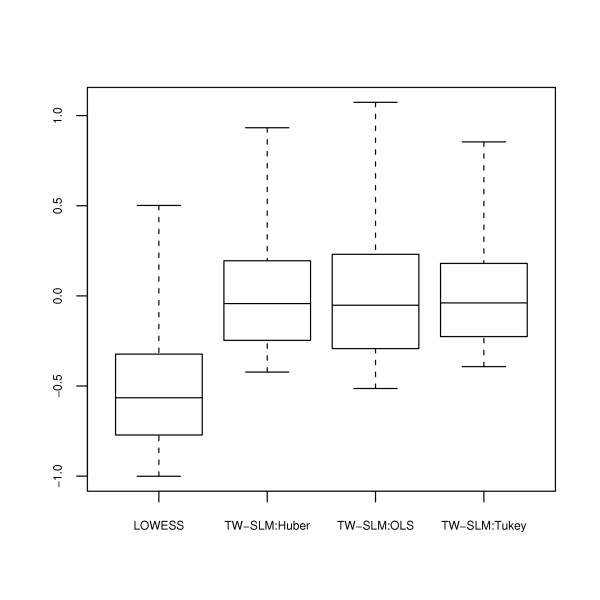
A boxplot for comparing biases among normalization methods for the case that 70% genes are all up-regulated (Table 4).

The data were normalized using the LOWESS normalization method and the robust TW-SLM with Tukey's robust weight function separately. Significance analysis was carried out for the normalized data for each method by comparing gene expression levels in the human placenta tissue relative to the universal reference. One sample t-test was used for the direct comparison and two-sample t-test was used for the indirect comparison. We used 10^-5 ^and 10^-3 ^as cutoff points for p-values to determine if clones are statistical significant or not. Consistency of estimated relative gene expression levels was compared between the direct design and the indirect design for each method. We also compared overlap between the LOWESS normalization method and the robust TW-SLM for each design. The results are presented in Figures 4 and 5.

We used 10^-5 ^as a cutoff point for p-values in Figure 4. Using the robust TW-SLM normalization and the t-tests, there are 2,907 genes with p-value less than 10^-5 ^in the direct comparison and 2,791 in the indirect comparison. There are 1,713 genes common in these two sets of genes with p-value less than 10^-5^, which account for about 59% (1713/2907) in the direct comparison and about 61% (1713/2791) in the indirect comparison.

In comparison, using the LOWESS normalization and the t-tests, there are 1,447 genes with p-value less than 10^-5 ^in the direct comparison and 1,045 in the indirect comparison. The number of overlapping genes with p-value less than 10^-5 ^is 467, which is around 32% (467/1447) in the direct comparison and about 44% (467/1045) in the indirect comparison. It is clear that the proposed method performs more consistent between the direct comparison and the indirect comparison.

We also examined overlap between the LOWESS and robust TW-SLM methods for the two comparisons. In the direct comparison, about 79% (1141/1447) of the genes found to be significant based on the LOWESS method are also found to be significant based on the robust TW-SLM method. But they only account for about 40% (1141/2907) of the significant genes detected based on the robust TW-SLM method. In the indirect comparison, about 71% (738/1045) of the significant genes based on the LOWESS method are also found to be significant based on the robust TW-SLM method. But they only account for about 26% (738/2791) significant genes detected based on the robust TW-SLM method.

In our analysis, we used background adjusted intensity values. How to adjust background is an important issue in microarray data analysis. To evaluate if background affects our conclusions, we repeated the comparison analysis without adjusting background for the intensity values in both channels, the results are presented in Tables 5 and 6. We see from these tables that the overall results are similar to those using background adjusted intensity values in normalization. This is what we would expect because of low and uniform distributed background noise in all arrays in this example (data description is not shown).

**Figure 8:**
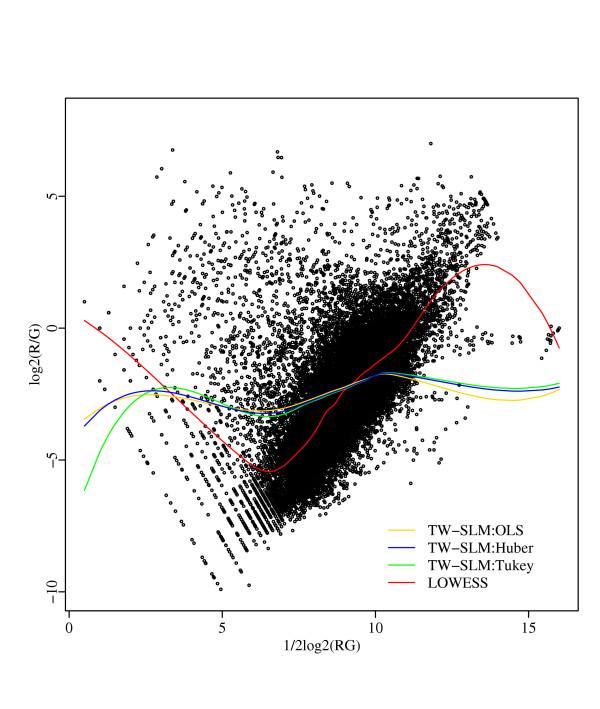
Slide-wise normalization curves based on different methods for one slide of the human placenta vs. the probe set hybridization in the example.

Therefore, the robust TW-SLM method yields more consistent results between the direct comparison and the indirect comparison with the human placenta and the universal human reference RNA samples. In addition, the robust TW-SLM method detects more significant genes for a given cutoff p-value. This makes sense biologically because most of the 7,042 genes specifically discovered from human placenta are expected to have differential expressions relative to the universal reference RNAs. We would expect that the similar comparison results will be got if we compare the TW-SLM using OLS or Huber's weight function with the LOWESS method because the normalization curves for the TW-SLMs (TW-SLM:OLS, TW-SLM:Huber, TW-SLM:Tukey) are similar, but all these three curves are different from the LOWESS normalization curve (Figure 8).

## Discussion

We have proposed a robust TW-SLM normalization method for cDNA microarray data. It is interesting to compare the proposed normalization method with the existing methods, such as the widely used LOWESS normalization proposed by Yang et al. (2001) [5] and further discussed by Tseng et al. (2001) [9]. In the LOWESS method, normalization is done separately by first fitting a separate curve for each slide through the R-I plot of log-intensity ratios versus log-intensity products. In comparison, the proposed method uses all the slides in estimating each normalization curve, using the gene effects *β*_*j *_as the thread linking these slides. In addition, in the proposed method, the normalization curves *φ*_*i *_and gene effects *β*_*j *_are estimated simultaneously. With this approach, there is no need to assume that the percentage of genes with differential expression levels is small or the expression levels of up- and down-regulated genes are symmetric, or when one of these assumptions is not satisfied, to use dye-swap normalization, which in turn requires the assumption that the two normalization curves are symmetric. (However, we note that dye-swap as a design strategy is useful to balance the experimental conditions and reduce bias due to different dye incorporation efficiencies.) An underlying condition required for the proposed method is independence of arrays, which is satisfied in a typical microarray experiment. Further theoretical conditions for the TW-SLM can be found in the paper by Huang et al. [17].

We have only considered the proposed robust TW-SLM method for the simple direct comparison design described in the Methods section. We can easily extend the method to more complicated designs. For example, we can adapt the proposed robust method to the TW-SLM that accommodates the design where a gene is printed multiple times. Such a design is helpful for improving the precision and for assessing the quality of an array using the coefficient of variation (Tseng et al. 2001 [9]). We can also adapt the robust TW-SLM to incorporate control genes with known concentration ratios in estimating the normalization curves. Model (1) can be easily extended to block-wise normalization by treating different blocks as separate arrays and normalization can be carried out as what we did here. Block-wise normalization considers spatial variation within an array. We did block-wise normalization on the data sets in the example and compared the results with that using the LOWESS method (Tables 5 and 6). The proposed method still outperforms the LOWESS method if we use block-wise normalization in this example.

## Conclusions

In our simulation studies, the proposed method performs better than the LOWESS normalization method in terms of MSEs of estimated gene effects in the simulation models we considered. Analysis of the probe set reference data set [19] shows that the proposed method yields more consistent results between the direct and indirect comparisons than the LOWESS normalization method. In addition, the proposed method is more sensitive in detecting differentially expressed genes than the LOWESS method. Therefore, we believe that the proposed robust TW-SLM method is a powerful alternative to the existing normalization methods. We have coded the proposed method in an **R **package which is available from the corresponding authors.

## Methods

We first describe the TW-SLM. For simplicity, we focus on the case of comparing two cell populations, in which two cDNA samples from the respective cell populations are competitively hybridized on the same array. Let *n *be the number of slides, and *J *be the number of genes in the study. Let *R*_*ij *_represent background corrected signal intensity from the Cy5 channel and *G*_*ij *_the background corrected signal intensity from the Cy3 channel, and let *y*_*ij *_= log_2_(*R*_*ij*_/*G*_*ij*_), *x*_*ij *_= (1/2) log_2_(*R*_*ij *_× *G*_*ij*_), for gene *j *on slide *i*. We assume that there is only one spot for each gene on each slide. The TW-SLM [17] is

*y*_*ij *_= *φ*_*i *_(*x*_*ij*_) + *β*_*j *_+ ∈_*ij*_, *i *= 1,..., *n*, *j *= 1,..., *J *    (1)

In this model, the observed log intensity ratio is decomposed into three components. The first component is *φ*_*i *_which is the intensity dependent normalization curve for slide *i*, the second component is *β*_*j *_which represents the relative expression value of the *j*th gene after normalization, the last one is the residual error term. Let 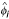 be a robust estimator of the *i*th normalization curve *φ*_*i *_based on this model described above. The normalized data are


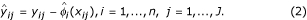


Huang et al. (2004) [17] considered the least squares method for estimating *φ*_*i *_and *β*_*j *_in the TW-SLM. However, it is well known that least squares estimates are not robust against outliers which often arise in microarray experiments. Therefore, we propose to use the robust method [20] for estimating *φ*_*i *_and *β*_*j*_. This is done by minimizing the objective function


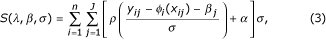


where *ρ *is an appropriately chosen function for robust estimation, *λ *is the collection of the coefficients in the spline representations of *φ*_*i *_described below, *σ *is the scale parameter, and *α *is a constant to be described below. We note here that estimation of *φ*_*i*_, *β*_*j *_are done jointly and uses data from all the arrays. This is different from the LOWESS normalization method in which estimation of normalization curves are done array by array.

We consider two *ρ *functions: Huber's *ρ *function and Tukey's biweight function. Huber's *ρ *function is


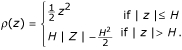


Tukey's biweight function is


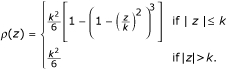


Two other usefull functions derived from *ρ*, *ψ *and *χ*, will be used repeatedly in the description of the algorithm below. They are defined as

*ψ*(*x*) = *ρ*'(*x*), *χ*(*x*) = *xψ*(*x*) - *ρ*(*x*).     (4)

The expressions of these functions are given in the Appendix. We choose commonly used constants in the literature for Huber's and Tukey's functions, i.e., *H *= 1.345 and *k *= 4.685. The influence of the choice of these constants on normalization methods is beyond the scope of this study.

We use the cubic B-splines [21,22] to approximate the normalization curves *φ*_*i*_. Specifically, let *b*_1_,..., *b*_*K *_be *K *B-spline basis functions. We approximate *φ*_*i *_by


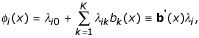


where **b**(*x*) = (1, *b*_1_(*x*),..., *b*_*K*_(*x*))' and *λ*_*i *_= (*λ*_*i*0_, *λ*_*i*1_,..., *λ*_*iK*_)'.

We estimate the parameters in model (1) by minimizing objective function (3) using an iterative procedure. Two steps, a location step and a scale step, will be used in the computation.

### Location step

We use the following vector and matrix notations in describing the location step:

**B**_**i **_= (**b**(*x*_*i*1_), **b**(*x*_*i*2_),..., **b**(*x*_*iJ*_))',

**y_i _**= (*y*_*i*1_, *y*_*i*2_,..., *y*_*iJ*_)'.

Let


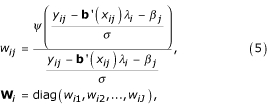


and let


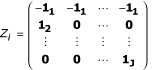


for *i *= 1,..., *n*. Given the scale parameter *σ*, 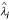 and 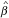 satisfy the equations:


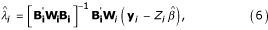



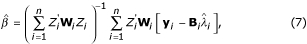


where 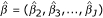 and 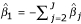 because of identifiability requirement in the TW-SLM. We can solve these equations iteratively to obtain 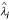 and 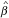. The derivations of these equations are given in the Appendix.

### Scale step

According to Huber's proposal [23], the estimation equation for *σ *is


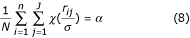


where *r*_*ij *_= *y*_*ij *_- **b**'(*x*_*ij*_) 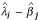, and *N *is the total number of observations in the data set. In general, equation (8) does not have an explicit solution. So we use the following updating equation to compute the estimated scale parameter *σ*,


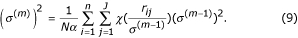


In order to obtain the consistent scale estimator at the normal distribution and obtain the classic estimates when using the least squares objective function, i.e., 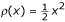, we used the constant suggested by Huber [23],


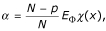


where *E*_Φ _denotes expectation with respect to the standard normal distribution function Φ.

The procedure described above is called an iterative reweighted least squares (IWLS) algorithm that is used in many non-least squares estimation problems. The implementation of the IWLS algorithm can be carried out using the following steps:

1. Initialize 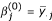 for *j *= 1,..., *J *and *σ*^(0) ^= 1, 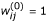, for *i *= 1,..., *n*, *j *= 1,..., *J*;

2. Calculate 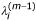 according to equation (6) given *β*^(*m*-1)^, *σ*^(*m*-1) ^and 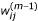 for *i *= 1,..., *n*, *j *= 1,..., *J*, *m *= 1,...;

3. Check convergence of *λ*_*i*_, *β*, and *σ*. If the convergence criteria is met, then stop, otherwise continue;

4. Update *σ*^(*m*) ^by equation (9) given 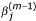, 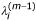, *σ*^(*m*-1)^, and 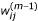, and set *σ*^(*m*-1) ^= *σ*^(*m*)^;

5. Calculate weight 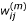 given *β*^(*m*-1)^, 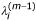 and *σ*^(*m*) ^according to equation (5), and set 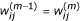;

6. Calculate *β*^(*m*) ^given 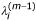
*σ*^(*m*-1) ^and 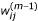 using equation (7), and set 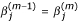;

7. Go to step 2 and iteratively update the estimators of parameters and the weights between steps 2 and 6 until convergence.

## Authors' contributions

DW devised and implemented the procedure described in the paper, drafted and finalized the manuscript. JH helped with writing and revising the manuscript. JH and MBS supervised and provided support for this work. HX and LM conducted the experiment that generated the data set used in the example.

## Appendix

### Derivation of 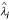 and 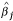


We derive estimation equations for location parameters presented in the **Methods **section in this appendix. Again the notations from the **Methods **section:

**b**(*x*) = (1, *b*_1_(*x*),..., *b*_*K*_(*x*))',

*λ*_*i *_= (*λ*_*i*0_, *λ*_*i*1_,..., *λ*_*iK*_)'.

*φ*_*i*_(*x*_*ij*_) can be approximated by a linear combination of B-spline basis functions, i.e. **b**'(*x*_*ij*_)*λ*_*i*_, where *b*_*k*_(*x*_*ij*_) is the *k*th B-spline basis function of *x*_*ij*_. Let *A *= (*a*_1_, *a*_2_,..., *a*_*n*_)', *C *= (*c*_1_, *c*_2_,..., *c*_*n*_)', and define


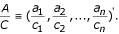


Given scale parameter *σ*, the first partial derivatives of *S*(*λ*, *β*, *σ*) (3) with respect to *λ *and *β *can be expressed in the matrix form as


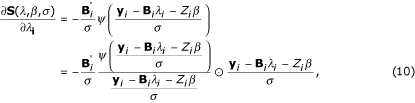



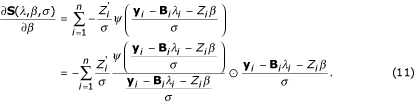


where **B_*i*_**; = (**b**(**x_i1_**), **b**(**x_i2_**),..., **b**(**x_iJ_**))', **y_i _**= (*y*_*i*1_, *y*_*i*2_,..., *y*_*iJ*_)', *ψ*(*x*) = *ρ*'(*x*). As defined in equation (5)


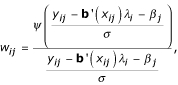


and

**W**_*i *_= diag(*w*_*i*1_, *w*_*i*2_,..., *w*_*iJ*_),

Plugging **W**_*i *_into equations (10) and (11) and setting them to zeros, and solving these two equations and yielding estimation equations for 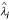 in equation (6) and 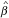 in equation (7). They are


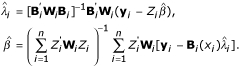


Let


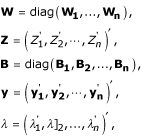


then equation (7) can also be expressed as


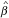
 = (**Z'WZ**)^-1 ^**Z'W**(**y **- **B**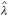).

The solution of (**Z'WZ**)^-1 ^can be explicitly calculates using the following matrix,


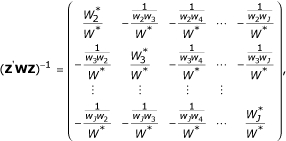


where 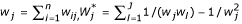, and 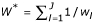 for *j *= 1,..., *J*. We can get the explicit solution of 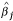 after doing some linear algebra. It is


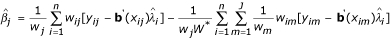


for *j *= 2,..., *J*. And 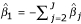 because of identifiability requirement in model (1).

### Derivation of scale parameter estimator 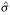


The *ψ *and *χ *functions derived from Huber's *ρ*(*z*) function are


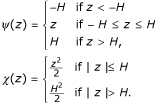


The related weight function has the form


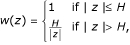


where H is a constant.

The constant *α *used in the scale step for Huber's robust estimation can be calculated as the following


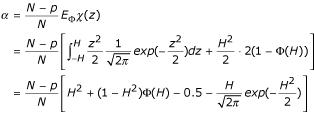


where *Φ *is the distribution function of the standard normal distribution, *N *is the total number of observations in the dataset, and *p *is the total number of parameters in the model.

The *ψ *and *χ *functions derived from Tukey's *ρ*(*z*) function are


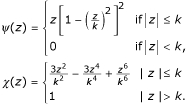


The associated weight function has the form


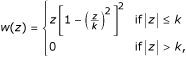


where *k *is a constant and the constant *a *in the scale step takes value


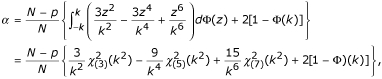


where 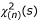 is the Chi-square probability function with *n *degrees of freedom evaluated at *s*.

When Tukey's weight function is used, equation (8) is solved directly for the estimator of *σ *instead of iteratively updating equation (9) in our **R **program. It can be shown that equation (8) for Tukey's *χ*(*z*) function has an unique real root. This real root is just the solution for the estimator of *σ*. Let n* be the total number of observations that satisfy the second case of Tukey's *χ *function, i.e. | *z *| >*k*, let J* be the total number of clones that satisfy the first case of the *χ*, i.e. | *z *| ≤ *k*. Replacing *z *by 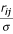 and plugging Tukey's *χ *into equation (8), we get


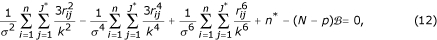


where


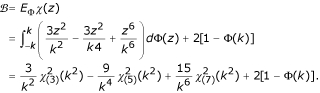


Let


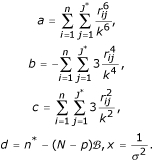


Then equation (12) becomes

*ax*^3 ^+ *bx*^2 ^+ *cx *+ *d *= 0.     (13)

Let *x *= *y *- *b*/3*a*, divided by *a *in the both sides of equation (13), and plugs *x *into equation (13), then we get the Cardan's cubic equation


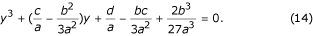


Let *p *= *c*/*a *- *b*^2^/3*a*^2^, *q *= *d*/*a *- *bc*/(3*a*^2^) + 2*b*^3^/(27*a*^3^), the above equation becomes

*y*^3 ^+ *py *+ *q *= 0.     (15)

The determinant for Cardan's equation (15) is


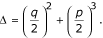


It can be shown that the determined function Δ must be positive. The first term in the determinant equation must be positive because of the square function and *q *cannot be zero. If we can show that the *p *is greater or equal to zero, then the Δ must be positive. Because


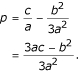


So the Δ is positive if only if 3*ac *- *b*^2 ^is non-negative. We can see that


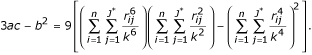


According to the Cauchy inequality [24], we have


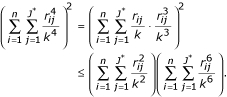


Therefore, the *p *must be non-negative and the Δ must be positive. Thus there is only one real root for equation (15), that is


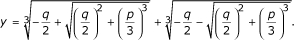


Then the solution for *σ *in equation (12) is


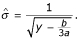


## Figures and Tables

**Table 1:** The mean square errors (MSE) of estimated gene expression levels (up:down = 9:1) for simulated cDNA microarray data with the R-I plots similar to Figure 2.

Percentage of DEG	Descriptive Statistics						
	
	Method	Mean	Minimum	25% Quantile	Median	75% Quantile	Maximum
1%	OLS	0.0837	0.0243	0.0474	0.0614	0.1074	1.7586
	Huber	0.0510	0.0106	0.0235	0.0312	0.0703	1.1750
	Tukey	0.0481	0.0101	0.0215	0.0291	0.0675	0.8684
	LOWESS	0.0849	0.0197	0.0485	0.0642	0.1085	1.7488
							
5%	OLS	0.0984	0.0197	0.0487	0.0648	0.1128	2.1413
	Huber	0.0605	0.0117	0.0246	0.0331	0.0740	1.5012
	Tukey	0.0556	0.0110	0.0226	0.0305	0.0712	1.2406
	LOWESS	0.0990	0.0234	0.0493	0.0677	0.1145	2.1275
							
10%	OLS	0.1198	0.0259	0.0519	0.0695	0.1244	1.7445
	Huber	0.0749	0.0118	0.0266	0.0371	0.0830	1.1705
	Tukey	0.0673	0.0111	0.0241	0.0340	0.0795	1.0206
	LOWESS	0.1196	0.0232	0.0514	0.0722	0.1264	1.7935
							
20%	OLS	0.1545	0.0239	0.0601	0.0855	0.1482	2.2914
	Huber	0.0983	0.0121	0.0322	0.0497	0.0994	1.4550
	Tukey	0.0854	0.0112	0.0285	0.0451	0.0932	1.1777
	LOWESS	0.1530	0.0244	0.0602	0.0900	0.1612	2.1958
							
40%	OLS	0.2099	0.0287	0.0835	0.1293	0.2086	2.3221
	Huber	0.1365	0.0155	0.0465	0.0827	0.1428	1.6918
	Tukey	0.1164	0.0135	0.0395	0.0735	0.1296	1.4402
	LOWESS	0.2220	0.0345	0.1153	0.1665	0.2491	2.8279

DEG: differentially expressed genes. OLS: the TW-SLM using the ordinary least squares.

Huber: the robust TW-SLM using Huber's weight function. Tukey: the robust TW-SLM using Tukey's weight function.

**Table 2:** Bias of estimated gene expression levels (up:down = 9:1) for simulated cDNA microarray data with the R-I plots similar to Figure 2.

Percentage of DEG	Descriptive Statistics						
	
	Method	Mean	Minimum	25% Quantile	Median	75% Quantile	Maximum
1%	OLS	0.0000	-1.2716	-0.0212	-0.0033	0.0154	1.2441
	Huber	0.0000	-1.0330	-0.0161	-0.0020	0.0115	0.9925
	Tukey	0.0001	-0.8617	-0.0153	-0.0014	0.0112	0.8462
	LOWESS	-0.0017	-1.2685	-0.0226	-0.0047	0.0137	1.2367
							
5%	OLS	0.0000	-0.8941	-0.0412	-0.0209	0.0019	1.4051
	Huber	0.0000	-0.7785	-0.0326	-0.0167	0.0007	1.1751
	Tukey	0.0000	-0.7195	-0.0299	-0.0146	0.0017	1.0581
	LOWESS	-0.0005	-0.8995	-0.0384	-0.0182	0.0008	1.3993
							
10%	OLS	0.0000	-1.1402	-0.0705	-0.0441	-0.0125	1.2574
	Huber	0.0000	-0.9240	-0.0578	-0.0353	-0.0096	1.0326
	Tukey	0.0000	-0.8245	-0.0521	-0.0313	-0.0064	0.9551
	LOWESS	-0.0050	-1.1332	-0.0666	-0.0429	-0.0207	1.2736
							
20%	OLS	0.0000	-1.4381	-0.1108	-0.0742	-0.0336	1.3336
	Huber	0.0000	-1.1569	-0.0927	-0.0607	-0.0247	1.1176
	Tukey	0.0000	-1.0430	-0.0841	-0.0540	-0.0175	0.9757
	LOWESS	-0.0325	-1.4180	-0.1352	-0.1009	-0.0634	1.2991
							
40%	OLS	0.0000	-1.4475	-0.1943	-0.1348	0.1870	1.3258
	Huber	0.0000	-1.2461	-0.1620	-0.1061	0.1474	1.1159
	Tukey	0.0000	-1.1429	-0.1460	-0.0907	0.1294	0.9800
	LOWESS	-0.1488	-1.6159	-0.3340	-0.2603	0.0182	1.1444

DEG: differentially expressed genes. OLS: the TW-SLM using the ordinary least squares.

Huber: the robust TW-SLM using Huber's weight function. Tukey: the robust TW-SLM using Tukey's weight function.

**Table 3:** The mean square errors (MSE) of estimated gene expression levels (up:down = 9:1) for simulated cDNA microarray data with the R-I plots similar to Figure 3.

Percentage of DEG	Descriptive Statistics						
	
	Method	Mean	Minimum	25% Quantile	Median	75% Quantile	Maximum
1%	OLS	0.0370	0.0116	0.0258	0.0323	0.0410	2.2092
	Huber	0.0248	0.0112	0.0195	0.0223	0.0255	1.7156
	Tukey	0.0238	0.0109	0.0191	0.0217	0.0247	1.5648
	LOWESS	0.0375	0.0119	0.0251	0.0321	0.0419	2.2162
							
5%	OLS	0.0489	0.0126	0.0265	0.0333	0.0422	1.5836
	Huber	0.0324	0.0110	0.0198	0.0228	0.0263	1.1765
	Tukey	0.0299	0.0108	0.0194	0.0222	0.0254	1.0278
	LOWESS	0.0493	0.0125	0.0255	0.0325	0.0429	1.6134
							
10%	OLS	0.0692	0.0124	0.0285	0.0359	0.0464	1.6907
	Huber	0.0455	0.0098	0.0210	0.0245	0.0288	1.1667
	Tukey	0.0404	0.0102	0.0204	0.0236	0.0276	1.0175
	LOWESS	0.0692	0.0119	0.0270	0.0349	0.0461	1.6846
							
20%	OLS	0.0961	0.0137	0.0324	0.0428	0.0570	1.8614
	Huber	0.0632	0.0124	0.0235	0.0282	0.0354	1.2969
	Tukey	0.0547	0.0127	0.0225	0.0266	0.0329	1.0525
	LOWESS	0.0950	0.0154	0.0325	0.0431	0.0580	1.8834
							
40%	OLS	0.1439	0.0147	0.0493	0.0665	0.1007	2.5021
	Huber	0.0960	0.0134	0.0330	0.0446	0.0673	1.9988
	Tukey	0.0821	0.0136	0.0305	0.0401	0.0602	1.6771
	LOWESS	0.1562	0.0187	0.0832	0.1121	0.1418	3.0480
							
*70%	OLS	0.1530	0.0138	0.0554	0.1146	0.1882	1.2717
	Huber	0.1040	0.0121	0.0366	0.0791	0.1267	0.9153
	Tukey	0.0901	0.0115	0.0337	0.0700	0.1098	0.7778
	LOWESS	0.4082	0.0350	0.1651	0.3563	0.6331	1.0816

DEG: differentially expressed genes. *: all DEG are up-regulated. OLS: the TW-SLM using the ordinary least squares.

Huber: the robust TW-SLM using Huber's weight function. Tukey: the robust TW-SLM using Tukey's weight function.

**Table 4:** Bias of estimated gene expression levels (up:down = 9:1) for simulated cDNA microarray data with the R-I plots similar to Figure 3.

Percentage of DEG	Descriptive Statistics						
	
	Method	Mean	Minimum	25% Quantile	Median	75% Quantile	Maximum
1%	OLS	0.0000	-0.8219	-0.0173	-0.0043	0.0094	1.4229
	Huber	0.0000	-0.6011	-0.0146	-0.0034	0.0080	1.2449
	Tukey	0.0000	-0.4942	-0.0137	-0.0031	0.0079	1.1431
	LOWESS	0.0017	-0.8382	-0.0155	-0.0018	0.0116	1.4261
							
5%	OLS	0.0000	-1.1839	-0.0307	-0.0151	0.0009	1.1853
	Huber	0.0000	-1.0325	-0.0258	-0.0118	0.0016	0.9497
	Tukey	0.0000	-0.9366	-0.0240	-0.0107	0.0024	0.8284
	LOWESS	0.0028	-1.1707	-0.0257	-0.0123	0.0015	1.1979
							
10%	OLS	0.0000	-1.2366	-0.0567	-0.0351	-0.0108	1.2074
	Huber	0.0000	-1.0440	-0.0477	-0.0297	-0.0078	1.0073
	Tukey	0.0000	-0.9654	-0.0444	-0.0270	-0.0056	0.9112
	LOWESS	0.0034	-1.2259	-0.0467	-0.0310	-0.0151	1.2333
							
20%	OLS	0.0000	-1.2168	-0.0922	-0.0677	-0.0368	1.3011
	Huber	0.0000	-0.9445	-0.0771	-0.0568	-0.0286	1.0866
	Tukey	0.0000	-0.8220	-0.0707	-0.0510	-0.0241	0.9765
	LOWESS	-0.0089	-1.2455	-0.0953	-0.0765	-0.0537	1.3045
							
40%	OLS	0.0000	-1.5360	-0.1722	-0.1230	0.1383	1.3821
	Huber	0.0000	-1.3680	-0.1451	-0.1030	0.1192	1.1008
	Tukey	0.0000	-1.2384	-0.1328	-0.0934	0.1114	0.9741
	LOWESS	-0.1418	-1.7047	-0.2937	-0.2553	-0.0205	1.2253
							
*70%	OLS	0.0000	-0.5137	-0.2925	-0.0519	0.2306	1.0736
	Huber	0.0000	-0.4227	-0.2466	-0.0429	0.1947	0.9327
	Tukey	0.0000	-0.3924	-0.2259	-0.0390	0.1802	0.8543
	LOWESS	-0.5203	-1.0007	-0.7719	-0.5651	-0.3230	0.5017

DEG: differentially expressed genes. *: all DEG are up-regulated. OLS: the TW-SLM using the ordinary least squares.

Huber: the robust TW-SLM using Huber's weight function. Tukey: the robust TW-SLM using Tukey's weight function.

**Table 5:** Consistency analysis with and without background adjustment under slide-wised and block-wised normalization strategies (the cutoff p-value is 10^-5^)

Normalization Strategy	Normalization Method	Direct comparison	Indirect comparison	Common genes^1^
**With background subtraction**				
Slide-wised	LOWESS	1447(32.27)^*a*^(78.85)^*b*^	1045(44.69) (70.62)	467
	TW-SLM	2907(58.93)(39.25)	2791(61.38)(26.44)	1713
	Common^2^	1141	738	-
Block-wised	LOWESS	1551(37.91)(76.47)	1464(40.16)(59.84)	588
	TW-SLM	2545(48.84) (46.60)	2267(54.83) (38.64)	1243
	Common^2^	1186	876	-
				
**Without background subtraction**				
Slide-wised	LOWESS	1240(37.98) (86.05)	1599(29.46) (77.42)	471
	TW-SLM	1924(72.77)(55.46)	3237(43.25)(38.25)	1400
	Common^2^	1067	1238	-
Block-wised	LOWESS	1357(46.13)(82.68)	2099(29.82) (69.46)	626
	TW-SLM	1904(59.51)(58.93)	2872(39.45) (50.77)	1133
	Common^2^	1122	1458	-

Common^1^: intersection between the direct and the indirect comparison given the same method.

Common^2^: intersection between the LOWESS and the TW-SLM given the same comparison.

^*a*^: consistency between the comparisons expressed as the percentage. 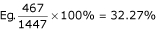.

^*b*^: the percentage of Common^2 ^for each method. 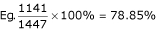.

**Table 6:** Consistency analysis with and without background adjustment under slide-wised and block-wised normalization strategies (the cutoff p-value is 10^-3^)

Normalization Strategy	Normalization Method	Direct comparison	Indirect comparison	Common genes^1^
**With background subtraction**				
Slide-wised	LOWESS	2563(44.52)^*a*^(79.05)^*b*^	2234(51.07)(73.23)	1141
	TW-SLM	4330(68.45) (46.79)	4055(73.09) (40.35)	2964
	Common^2^	2026	1636	-
Block-wised	LOWESS	2731(49.40)(78.58)	2700(49.96) (66.74)	1349
	TW-SLM	4085(61.32)(52.53)	3645(68.72) (49.44)	2505
	Common^2^	2146	1802	-
				
**Without background subtraction**				
Slide-wised	LOWESS	2440(52.50) (82.99)	3024(42.36) (79.27)	1281
	TW-SLM	3615(77.01)(56.02)	4400(63.27) (54.48)	2784
	Common^2^	2025	2397	-
Block-wised	LOWESS	2694(57.24) (79.62)	3495(44.12)(74.91)	1542
	TW-SLM	3530(67.39) (60.76)	4190(56.79)(62.48)	2379
	Common^2^	2145	2618	-

Common^1^: intersection between the direct and the indirect comparison given the same method.

Common^2^: intersection between the LOWESS and the TW-SLM given the same comparison.

^*a*^: consistency between the comparisons expressed as the percentage. 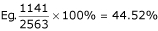.

^*b*^: the percentage of Common^2 ^for each method. 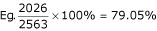.
